# Driver Fatigue Detection Using Heart Rate Variability Features from 2-Minute Electrocardiogram Signals While Accounting for Sex Differences

**DOI:** 10.3390/s24134316

**Published:** 2024-07-03

**Authors:** Chao Zeng, Jiliang Zhang, Yizi Su, Shuguang Li, Zhenyuan Wang, Qingkun Li, Wenjun Wang

**Affiliations:** 1 College of Information Science and Engineering, Henan University of Technology, Zhengzhou 450001, China; zengchao@haut.edu.cn (C.Z.);; 2 Hami Vocational and Technical College, Hami 839001, China; 3 School of Vehicle and Mobility, Tsinghua University, Beijing 100084, China; 4 School of Automation Engineering, University of Electronic Science and Technology of China, Chengdu 611731, China; 5 Beijing Key Laboratory of Human-Computer Interaction, Institute of Software, Chinese Academy of Sciences, Beijing 100190, China

**Keywords:** driving fatigue, ECG, 2 min HRV features, sex difference, decision tree

## Abstract

Traffic accidents due to fatigue account for a large proportion of road fatalities. Based on simulated driving experiments with drivers recruited from college students, this paper investigates the use of heart rate variability (HRV) features to detect driver fatigue while considering sex differences. Sex-independent and sex-specific differences in HRV features between alert and fatigued states derived from 2 min electrocardiogram (ECG) signals were determined. Then, decision trees were used for driver fatigue detection using the HRV features of either all subjects or those of only males or females. Nineteen, eighteen, and thirteen HRV features were significantly different (Mann–Whitney U test, *p* < 0.01) between the two mental states for all subjects, males, and females, respectively. The fatigue detection models for all subjects, males, and females achieved classification accuracies of 86.3%, 94.8%, and 92.0%, respectively. In conclusion, sex differences in HRV features between drivers’ mental states were found according to both the statistical analysis and classification results. By considering sex differences, precise HRV feature-based driver fatigue detection systems can be developed. Moreover, in contrast to conventional methods using HRV features from 5 min ECG signals, our method uses HRV features from 2 min ECG signals, thus enabling more rapid driver fatigue detection.

## 1. Introduction

Driver fatigue caused by inadequate sleep, circadian rhythm disruption, and prolonged mental activity has become a primary contributor to traffic accidents and a major public health concern. Statistics indicate that drowsy driving accounts for up to 20–25% of all motor vehicle crashes and is responsible for thousands of deaths each year [1]. Driver fatigue leads to impaired reaction times, reduced vigilance, information processing deficits, and lapsed attention, which are all extremely dangerous while operating a vehicle. Detecting driver fatigue through accurate, timely methods is therefore essential for preventing these often-fatal incidents. With the development of more rapid and reliable approaches for monitoring fatigue levels while driving, drivers could be alerted of potential issues, enabling interventions before disastrous accidents occur. This highlights the need for research focused on real-time driver fatigue detection based on psychophysiological metrics.

Heart rate variability (HRV) is a promising psychophysiological indicator of driver fatigue that can be unobtrusively measured in real-world settings [2,3]. HRV refers to subtle variations in the intervals between successive heartbeats, which reflect dynamic changes in autonomic nervous system activity [2]. The autonomic nervous system, which is composed of sympathetic and parasympathetic branches, regulates involuntary processes such as heart rate to maintain homeostasis and adapt to situational demands [4,5]. As electrocardiography (ECG) can reflect the electrical activity of a beating heart, HRV data derived from ECG could be used for investigations of cardiac autonomic modulation and of the interactions between sympathetic and parasympathetic activity. Frequency domain HRV markers, such as the power in the high-frequency (HF) and low-frequency (LF) bands, provide information on parasympathetic and sympathetic activity, respectively [6,7]. Moreover, time-domain HRV markers and nonlinear HRV markers indicate different driver states [4,8,9]. HRV is influenced by various factors, including age, sex, cardiorespiratory fitness level, circadian rhythms, and psychological stress [10]. Moreover, the optimal duration for HRV analysis depends on the specific marker and application context. For instance, standard short-term HRV assessments involve 5 min electrocardiogram (ECG) recordings, but some markers can be detected based on 1–2 min segments [11]. Research indicates that certain HRV features are altered while driving in a fatigued state; these include changes in the LF/HF ratio and complexity metrics [12,13].

While HRV shows potential for detecting driver fatigue, prior works have several limitations. In standard HRV analysis, 5 min ECG recordings are acquired to ensure the stability of the obtained markers; however, these recordings delay the detection of fatigue onset during driving [14]. The Task Force guidelines on HRV measurement [2], interpretation, and clinical use suggest that 5 min is the optimal duration for short-term HRV analysis, while 2 min is the acceptable minimum duration (although certain features, such as very-low-frequency (VLF) domain features, may lose physiological significance with this duration). Although some studies have explored the extraction of limited HRV features from shorter ECG signals (e.g., 60 s or even 30 s) [15,16,17,18], these methods have not been widely used due to their limited feature extraction potential. In driver fatigue detection, the ability to detect fatigue using shorter ECG signals is advantageous, enabling faster alerts and intervention for drivers. If fatigue can be detected using shorter ECG signals, quicker warnings and interventions to prevent potential accidents could be implemented.

The relationship between HRV and driver fatigue is complex and influenced by various factors related to the individuals being studied. One important factor affecting HRV features is sex. Research has consistently shown that sex considerably impacts HRV characteristics [19,20,21,22]. Studies have established that sex influences HRV features, with females exhibiting greater parasympathetic activity markers such as HF power [23,24,25]. However, in driver fatigue, few studies have comprehensively examined sex-specific HRV features during wakefulness and fatigue on the basis of 2 min ECG signals. Furthermore, no studies have built driver fatigue detection models based on sex-specific HRV features, accounting for the influence of sex on the relationship between HRV features and fatigue.

To address these research gaps, in the present study, we aimed to extract comprehensive sets of time-domain, frequency-domain, and nonlinear HRV features specific to each sex from 2 min ECG signals. We adopted a decision tree as a classification method because this approach can contend with multivariate nonlinear features and outliers and demonstrates stronger interpretative abilities than other popular classifiers such as support vector machines and artificial neural networks [26].

The objectives of this study are to investigate the relationship between HRV features and driver fatigue considering sex-specific characteristics and to develop sex-specific driver fatigue detection models using decision tree classifiers. The remainder of this paper is organized as follows. Section 2 presents the methodology employed in this study. Section 3 and Section 4 present the results and discussion. Finally, the conclusions are presented in Section 5.

## 2. Materials and Methods

### 2.1. Data Acquisition and Processing

**Figure 1:**
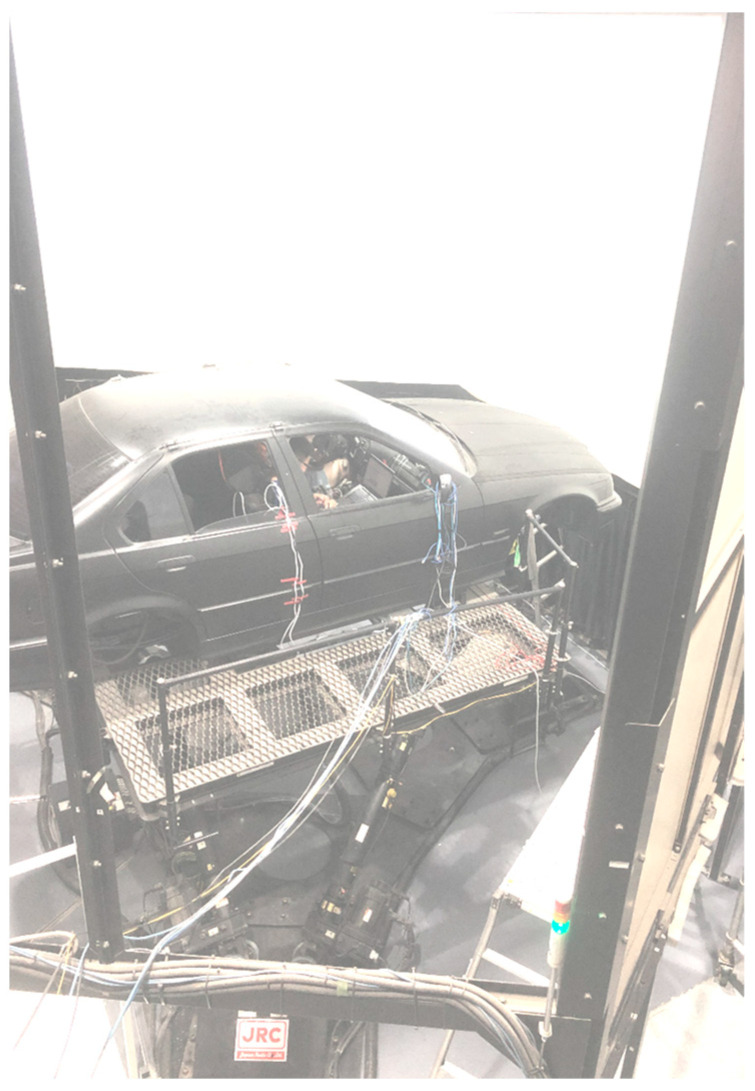
High-fidelity driving simulator.

ECG and facial expression data were collected from nine male and nine female college students with legal driver licenses during simulated driving experiments and further processed. The age of the subjects was 25.95 ± 2.67 years, and the BMI of the subjects was 21.03 ± 2.87 kg/m^2^. Subjects were asked to refrain from consuming caffeine, alcohol, and tea on the testing day. The experiments, which involved a straight highway driving scenario, were performed in a driving simulator (Figure 1) with six degrees of freedom. The duration of the experiments was 60 min, and during that period, the subject drove a car in a virtual environment with no interruptions, and there were no other cars in the scenario.

From videos of each driver’s facial expressions, five well-trained experts evaluated the driver’s mental state every minute as ‘alert’, ‘fatigued’, or ‘very fatigued’ according to the criteria in Table 1. Then, the 60 min videos were divided into 59 two-minute segments, with 50% overlap with the start and end of the subsequent and previous segments, respectively. For each segment, if both minutes were evaluated by three or more experts as ‘alert’, the segment was marked as an alert segment, and if both minutes were evaluated by three or more experts as ‘fatigued’ or ‘very fatigued’, the segment was marked as a fatigued segment; otherwise, the segment was marked as a discordant segment. With this approach, we obtained 272 alert segments and 638 fatigued segments for the following analyses, as well as 152 discordant segments, which were excluded from further analysis.

**Figure 2:**
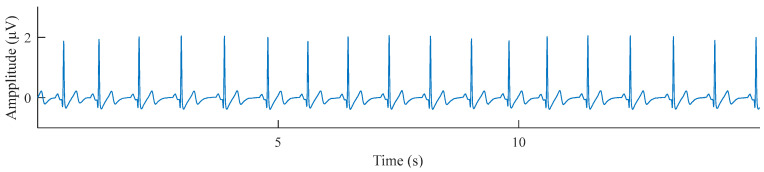
Illustration of the acquired ECG signal.

ECG signals (Figure 2) were acquired at a sampling frequency of 1 kHz with the modified lead II configuration adopted by the Biopac MP 150 system (Biopac Inc., Goleta, CA, USA). For all alert and fatigued video segments, the synchronously collected two-minute ECG segments were also extracted from the 60 min ECG signal. The R peaks were detected, and the HRV features listed in Table 2 were extracted using Kubios 3.0.2 (Kubios Oy, Kuopio, Finland) following the guideline of the Task Force [2].

In summary, during the 60 min simulated driving experiments, facial expressions and ECG signals were synchronously recorded, and then both recordings were divided into 59 successive two-minute segments. Each segment overlapped with the adjacent segments by 50%. Then, HRV features were extracted from each ECG segment, and the driver’s mental state was determined according to each facial expression segment for the following analysis. Interested readers can refer to Refs. [9,27] for a detailed description of the experimental design, fatigue assessment approach used for the video segments, and HRV feature extraction method.

### 2.2. Statistical Analysis

We calculated the means, standard deviations, medians, 1st quartiles (Q1), and 3rd quartiles (Q3) to describe the distributions of HRV features for subjects in the alert and fatigued states. The subjects were initially combined into one group, then divided into male and female groups. Mann–Whitney U tests were used to investigate the statistical significance of differences in features between subjects in the alert state and fatigued state in the combined, male, and female groups. Statistical analyses were performed with MATLAB version R2016b (The MathWorks Inc., Natick, MA, USA).

### 2.3. Classification and Performance Measurement

**Figure 3:**
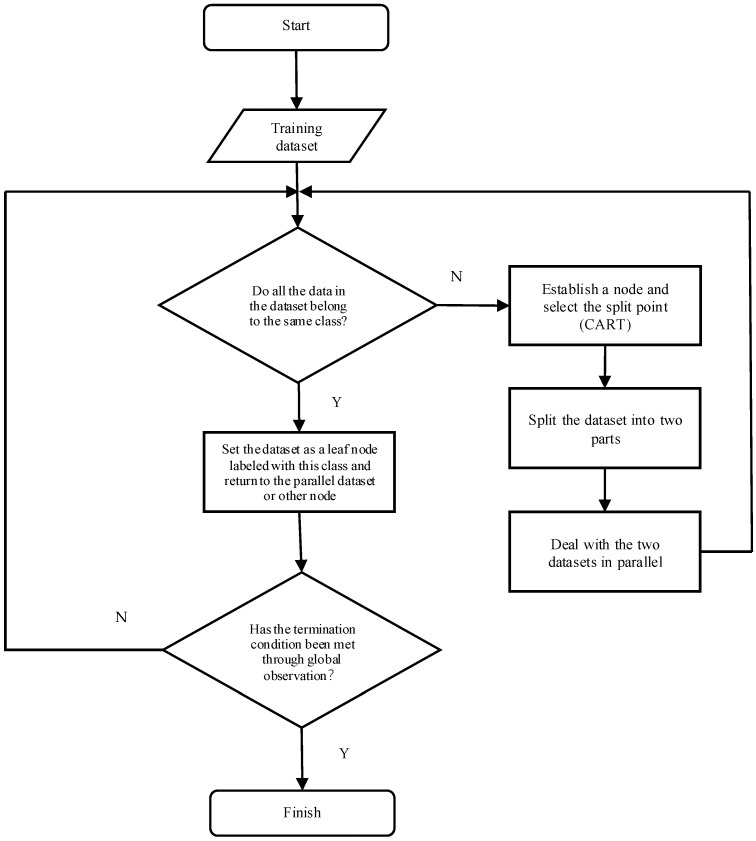
Illustration of the decision tree construction process.

We adopted the decision tree algorithm as the classification method. ID3, C4.5, and CART are classic decision tree algorithms. Among them, the ID3 algorithm is a relatively early decision tree algorithm that cannot handle continuous-valued features, and the information may be biased because it favors selecting features with more values. The C4.5 algorithm is an improved approach proposed to address the issues of the ID3 algorithm. However, this algorithm introduces gain ratio and pruning, allowing it to be too flexible when applied to practical classification problems. The CART algorithm introduces the Gini coefficient to optimize computations; thus, this algorithm has simpler splitting rules, more unified splitting criteria, more efficient computations, shorter training times, and stronger robustness and stability than the C4.5 algorithm. Therefore, the CART decision tree is used as the classification method in this paper. The construction process of the decision tree is shown in Figure 3. For each categorizable dataset, different from the ID3 and C4.5 algorithms, the CART decision tree algorithm establishes a node and selects a feature with the smallest Gini coefficient as the split point from the given feature combination to split the dataset into two parts. Then, the model iterates until all nodes contain either the same class or the number of samples in the node is less than a certain threshold. In the ID3 and C4.5 algorithms, the selection of decision tree nodes is performed based on the entropy model. The entropy model involves many logarithmic operations to represent the classification performance of the current node of the decision tree which could slow the computation and affect the convergence of the model. The CART algorithm solves this problem by using the Gini coefficient instead of the entropy model to represent the performance of the node. The smaller the Gini coefficient is, the better the current node classification performance is.

In this study, two-minute HRV features of all subjects were divided into a training set and a test set at a ratio of 8:2. The training set was used to establish the classifier, and the test set was used to evaluate the performance of the classifier by computing the sensitivity (SEN), positive predictive value (PPV), and accuracy (ACC) using the following formulae:

(1) $$\mathrm{SPE}=\frac{\mathrm{TN}}{\mathrm{TN}+\mathrm{FP}},$$

(2) $$\mathrm{SEN}=\frac{\mathrm{TP}}{\mathrm{TP}+\mathrm{FN}},$$

(3) $$\mathrm{PPV}=\frac{\mathrm{TP}}{\mathrm{TP}+\mathrm{FP}},$$

(4) $$\mathrm{ACC}=\frac{\mathrm{TP}+\mathrm{TN}}{\mathrm{TP}+\mathrm{TN}+\mathrm{FP}+\mathrm{FN}}$$

where TP is the number of ECG segments collected during the fatigued state that were correctly detected, TN is the number of ECG segments collected during the alert state that were correctly detected, FP is the number of ECG segments collected during the alert state that were incorrectly labeled as the fatigued state, and FN is the number of ECG segments collected during the fatigued state that were incorrectly labeled as the alert state.

The so-called exhaustive search method [7] was adopted for feature selection. All HRV feature subsets with two, three, and four features were listed, and decision tree models were established, with their performance evaluated. The number of combinations of two, three, and four HRV features chosen from twenty HRV features was 190, 1140, and 4845, respectively.

## 3. Results

Table 3 presents both the descriptive statistics and the results of the Mann–Whitney U test for the twenty HRV features of the drivers in the alert and fatigued states. Nineteen of the twenty HRV features showed significant (*p* < 0.01) differences between the two mental states. Compared with being alert, being fatigued led to significantly increased $M_{RR}$, $D_{RR}$, $D_{RMS}$, $N_{NN15}$, $p_{NN15}$, $P_{LF(abs)}$, $P_{HF(abs)}$, $P_{LF(nu)}$, $r_{LF/HF}$, $P_{tot(abs)}$, $L_{SD1}$, $L_{SD2}$, $r_{SD2/SD1}$, $\alpha_{1}$, and $D_{2}$ values and significantly decreased $M_{HR}$, $P_{HF(nu)}$, $E_{AP}$, and $E_{Samp}$ values.

**Figure 4:**
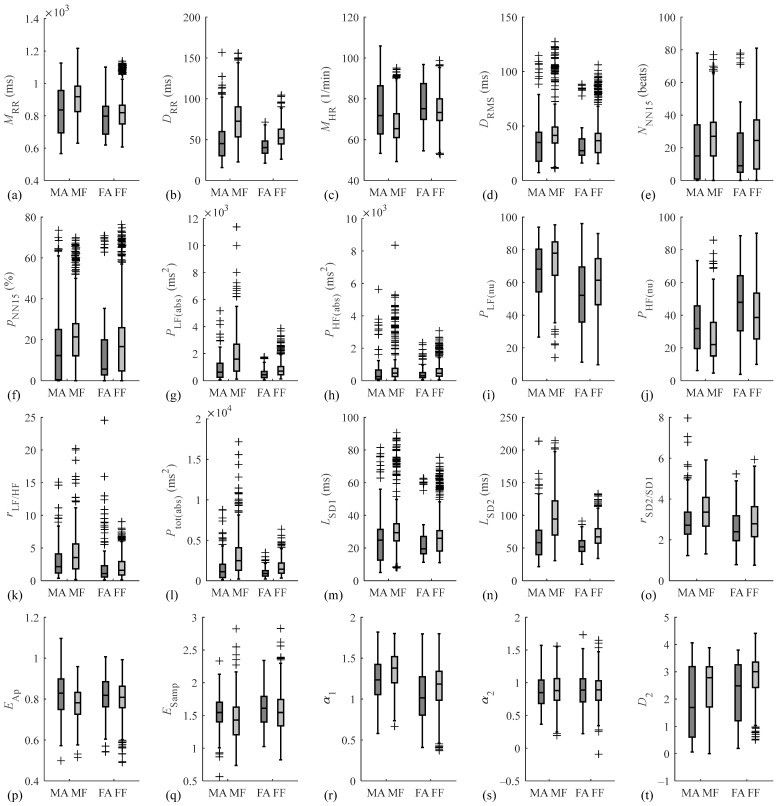
Box plots of HRV features of drivers in different mental states and drivers of different sexes. (MA denotes the male group in the alert state; MF denotes the male group in the fatigued state; FA denotes the female group in the alert state; and FF denotes the female group in the fatigued state).

The drivers were then divided into male and female groups. In the male group, as shown in Table 4, the significant differences in all the features between the alert and fatigued states were essentially the same as those in the sex-independent group, except for $D_{2}$, which significantly increased from the alert state to the fatigued state in the sex-independent group but was not significantly different between the two states in the male group. However, in the female group, as shown in Table 5, $D_{2}$ was significantly different between the two mental states. Moreover, $E_{AP}$ and $E_{Samp}$, which were significantly decreased from the alert state to the fatigued state in the sex-independent group and male group, were not significantly different in the female group. $\alpha_{2}$ was the only feature that was not significantly different between the two mental states in all three groups. Box plots of the HRV features of drivers in different mental states and drivers of different sexes are shown in Figure 4.

We then compared the results of our previous studies [9,27], in which 5 min ECG segments were employed for HRV feature extraction, and the findings of this study to identify HRV features that differed significantly between the two mental states, as shown in Table 6. In particular, we focused on the change tendency of the HRV features between the alert and fatigued states. In the sex-independent, male, and female groups, twelve, eight, and six of the eighteen HRV features differed significantly between the two mental states in both our previous study and this study. Moreover, these features were considered to have the same change tendency if *p* < 0.01. Regarding the differences between the studies in terms of significantly different HRV features, in all cases, no significant difference in HRV features between the two mental states was shown in our previous study, while a significant difference was identified in the present study.

**Figure 5:**
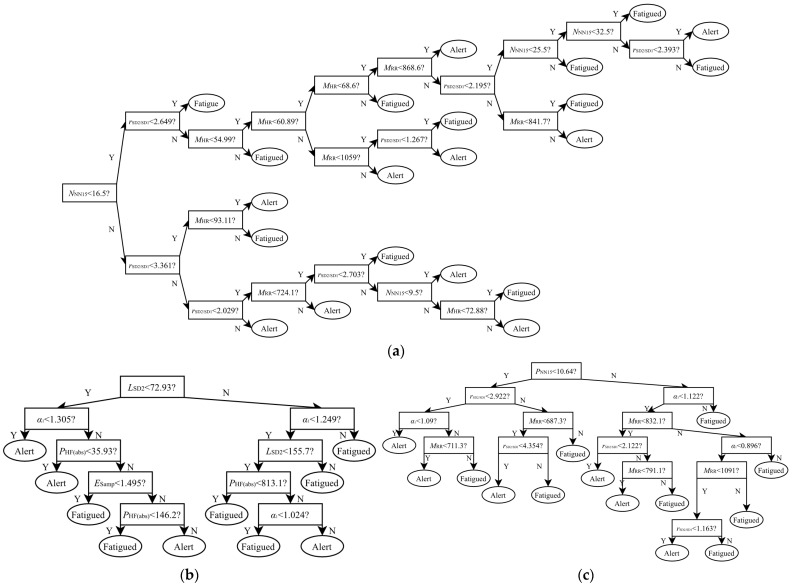
Decision tree and HRV features based driver fatigue classifier for the (**a**) sex-independent group; (**b**) male group; and (**c**) female group.

Table 7 shows the performance of the classifiers that achieved the highest accuracy based on subsets of two to four HRV features. For the sex-independent group, with subsets consisting of two features ($p_{NN15}$ and $r_{SD2/SD1}$), three features ($M_{RR}$, $N_{NN15}$, and $r_{SD2/SD1}$), and four features ($M_{RR}$, $M_{HR}$, $N_{NN15}$, and $r_{SD2/SD1}$), the models achieved the highest classification accuracies of 83.5%, 86.3%, and 86.3%, respectively. The results of the driver fatigue detection model with four features for this group are shown in Figure 5a. For the male group, with subsets consisting of two features ($M_{RR}$ and $r_{SD2/SD1}$), three features ($P_{HF(nu)}$, $\alpha_{2}$, and $D_{2}$) and four features ($P_{HF(abs)}$, $L_{SD2}$, $E_{Samp}$, and $\alpha_{1}$), the models achieved the highest classification accuracies of 92.0%, 93.5%, and 94.8%, respectively. The results of the driver fatigue detection model with four features for this group are shown in Figure 5b. For the female group, with subsets consisting of two features ($D_{RR}$ and $P_{LF(abs)}$), three features ($M_{RR}$, $p_{NN15}$, and $\alpha_{1}$), and four features ($M_{RR}$, $p_{NN15}$, $r_{SD2/SD1}$, and $\alpha_{1}$), the models achieved the highest classification accuracies of 88.5%, 90.7%, and 92.0%, respectively. The results of the driver fatigue detection model with four features for this group are shown in Figure 5c.

## 4. Discussion

In this study, we compared the HRV features of drivers in alert and fatigued states considering sex differences. The experiments were performed on a driving simulator, and the drivers’ fatigued state was induced by monotonous driving. Based on the data collected, Mann–Whitney U tests were conducted considering driver state and sex as group factors, and decision tree models were constructed to identify the drivers’ fatigued state using different feature combinations.

Table 6 presents a comparison of the Mann–Whitney U test results between this study and our team’s previous studies based on the same ECG dataset using 5 min ECG signals [9,27]. When sex differences both were and were not considered as one of the group factors, the direction of changes in the features in this study was consistent with that found in our previous study except for those features without significant difference between the two mental states in one of the studies. In other words, no controversial results were identified regarding the change direction from alert to fatigued between HRV features extracted from 2 min and 5 min ECG. In addition, some features that showed no significant differences between states in our previous study showed significant differences in this study, indicating that, compared with 5 min ECG signals, 2 min ECG signals were comparable or more sensitive measurements.

Glaucylara et al. [19] showed that short-term ECG signals were not suitable for studying VLF bands, so the frequency domain features in this paper included only HF and LF bands. According to Mietus et al. [28], who utilized a range of $p_{NNx}$ values from $p_{NN4}$ to $p_{NN100}$ to differentiate between sleeping and waking states in 72 healthy subjects, the differences between the comparison groups were consistently observed by using threshold values substantially less than 50 ms. Their results confirmed that $p_{NN12}$ enabled better distinction between sleeping and waking groups than $p_{NN50}$. Nagy and Jobbágy [29] comprehensively investigated the changes in HRV during stress by employing multiple features, including $p_{NN15}$ and $p_{NN50}$. The results revealed that $p_{NN15}$ showed more significant differences than $p_{NN50}$. Therefore, according to these previous works, we aimed to utilize features $p_{NN15}$ and $N_{NN15}$ to analyze the fatigued state. According to previous standards [2], a 2 min interval is the shortest interval within which driver fatigue can be detected. Consequently, driver fatigue detection based on 2 min ECG signals has the potential to identify a driver’s fatigue state in the shortest possible time.

The classification results based on the decision tree model corroborate the findings of the statistical analysis. In each analysis, the sex-specific classification models consistently obtained greater accuracy than the sex-independent models. The results suggest that a driver fatigue detection system that accounts for sex differences could achieve enhanced classification accuracy. In addition, the comparison reveals that the classification accuracy consistently increased as the number of considered features increased. However, as the number of features used in the classifier increased, the risk of classifier overfitting also increased.

While our study’s results indicate the effectiveness of some features in driver fatigue detection, there is ongoing debate regarding the performance of certain features in different studies. This controversy may arise from various factors, including variations in study designs, differences in sample sizes, diverse data processing methods, and the wide range of driving conditions considered. Therefore, we emphasize the need for further research into the physiological mechanisms associated with these features and the relationships between these features to comprehensively address these inconsistencies and improve our understanding of the role of HRV features in driver fatigue detection. Due to the complexity and large number of controlled variables, conducting experiments that account for all influencing factors remains challenging. In this study, various control variables, including circadian rhythms, respiratory frequency, human–machine interfaces, and substances such as drugs and alcoholic beverages, were not accounted for in the experiments. In conclusion, future research should consider more control variables to improve the generalizability of the results. Moreover, due to the constraints posed by the experimental conditions, performing such experiments with real road scenarios under similar conditions remains unfeasible.

## 5. Conclusions

In conclusion, the results of the present study suggest that HRV features derived from 2 min ECG recordings can be used to characterize drivers’ mental states based on both significant differences and classification performance. In the sex-independent group of drivers, nineteen of the twenty HRV features showed significant differences between the two mental states, and a decision tree-based classifier achieved an accuracy of 86.3% in detecting driver fatigue using a subset of HRV features.

In addition, our study demonstrated that HRV features differ between sexes, which could be used to characterize the mental states of drivers. (1) Eighteen HRV features (except for $\alpha_{2}$ and $D_{2}$) were significantly different between the two mental states for males, and seventeen features (except for $E_{AP}$, $E_{Samp}$ and $\alpha_{2}$) were significantly different for females. (2) The developed classifiers always achieved better fatigue detection accuracy for the male and female groups than for the sex-independent group when the number of HRV features in the subset ranged from 2 to 4. (3) For the best classifiers, when the number of HRV features in the subset ranged from 2 to 4, the selected features for both the male and female groups were always different.

## Figures and Tables

**Table 1:** Subjective evaluation criteria for drivers’ mental state assessment.

Mental State	Facial Feature Description
Alert	Eyes open normally, blinking rapidly, active eye state, mentally focused, maintains attention on the external world, head upright, rich facial expression.
Fatigued	Tendency to close the eyes, eyes are less open, blinking longer and slower, less active eye state, dull gaze, yawning, deep breathing, sighing, swallowing movements, movements to resist fatigue such as squeezing the eyes, shaking the head, and scratching the face, less focus on the environment.
Very Fatigued	Severe tendency for eyes to close, eyes half-closed, eyelids become heavy and cannot be opened, longer periods of persistent eye closure, nodding, head tilted, loss of ability to continue driving.

**Table 2:** List of HRV features studied.

Method	Features	Description	Unit
Time domain	$M_{RR}$	Mean RR interval	ms
	$D_{RR}$	Standard deviation of the RR interval	ms
	$M_{HR}$	Mean heart rate	1/min
	$D_{RMS}$	Square root of the mean squared differences between successive RR intervals	ms
	$N_{NN15}$	Number of successive RR interval pairs that differ by more than 15 ms	beats
	$p_{NN15}$	$N_{NN15}$ divided by the total number of RR intervals	%
Frequency domain	$P_{LF(abs)}$	Absolute power of the LF band	ms^2^
	$P_{HF(abs)}$	Absolute power of the HF band	ms^2^
	$P_{LF(nu)}$	Power of the LF band in normalized units	n.u.
	$P_{HF(nu)}$	Power of the HF band in normalized units	n.u.
	$r_{LF/HF}$	Ratio of the LF band power to the HF band power	−
	$P_{tot(abs)}$	Absolute power of all three bands	ms^2^
Nonlinear	$L_{SD1}$	The standard deviation of the PP perpendicular to the line of identity	ms
	$L_{SD2}$	The standard deviation of the PP along the line of identity	ms
	$r_{SD2/SD1}$	Ratio of the parameters of the short axis to the long axis of the PP	−
	$E_{AP}$	Approximate entropy	−
	$E_{Samp}$	Sample entropy	−
	$\alpha_{1}$	Short-term fluctuation slope in detrended fluctuation analysis	−
	$\alpha_{2}$	Long-term fluctuation slope in detrended fluctuation analysis	−
	$D_{2}$	Correlation dimension	−

**Table 3:** Descriptive statistics and results of Mann–Whitney U tests of the HRV features of drivers with different mental states in the sex-independent group.

Features	Alert (N = 272)					Fatigued (N = 638)					Alert vs. Fatigued
	Mean	SD	Q1	Med	Q3	Mean	SD	Q1	Med	Q3	Mann–Whitney U Test
$M_{RR}$	801.4	134.8	687.6	800.6	870.7	869.9	139.3	766.0	845.6	926.9	*p* < 0.01
$D_{RR}$	45.26	19.96	32.91	40.95	52.22	64.04	24.85	46.09	59.50	76.06	*p* < 0.01
$M_{HR}$	76.94	12.56	68.91	74.94	87.27	70.72	11.16	62.31	70.21	78.33	*p* < 0.01
$D_{RMS}$	34.27	20.61	21.16	27.51	41.04	43.30	23.71	25.86	39.40	45.79	*p* < 0.01
$N_{NN15}$	19.53	21.47	3.000	9.000	31.50	28.07	21.43	8.000	26.50	36.00	*p* < 0.01
$p_{NN15}$	15.05	18.52	1.824	5.861	23.29	22.55	19.90	5.385	19.70	26.67	*p* < 0.01
$P_{LF(abs)}$	729.1	741.6	243.4	494.4	879.7	1411	1343	561.1	924.8	1869	*p* < 0.01
$P_{HF(abs)}$	518.3	697.1	162.4	288.6	614.9	749.6	915.7	273.3	464.5	761.8	*p* < 0.01
$P_{LF(nu)}$	60.32	20.19	44.64	62.01	76.65	65.79	19.04	54.54	69.88	81.15	*p* < 0.01
$P_{HF(nu)}$	39.56	20.17	23.26	37.97	55.23	34.10	18.99	18.61	30.10	45.36	*p* < 0.01
$r_{LF/HF}$	2.803	3.237	0.8080	1.633	3.295	3.164	2.839	1.200	2.322	4.354	*p* < 0.01
$P_{tot(abs)}$	1332	1331	513.2	932.7	1730	2371	2098	1045	1744	2899	*p* < 0.01
$L_{SD1}$	24.32	14.65	15.01	19.52	29.13	30.74	16.85	18.35	27.98	32.54	*p* < 0.01
$L_{SD2}$	58.54	25.60	43.42	53.11	67.43	83.23	33.21	61.18	76.43	100.1	*p* < 0.01
$r_{SD2/SD1}$	2.776	1.046	2.082	2.578	3.334	3.106	1.061	2.365	3.034	3.805	*p* < 0.01
$E_{AP}$	0.818	0.101	0.755	0.827	0.894	0.789	0.086	0.739	0.797	0.849	*p* < 0.01
$E_{Samp}$	1.575	0.275	1.402	1.577	1.736	1.500	0.322	1.283	1.487	1.696	*p* < 0.01
$\alpha_{1}$	1.145	0.302	0.905	1.117	1.362	1.238	0.278	1.075	1.260	1.436	*p* < 0.01
$\alpha_{2}$	0.879	0.256	0.698	0.873	1.048	0.893	0.247	0.732	0.888	1.037	*p* = 0.2614
$D_{2}$	2.107	1.240	0.782	2.332	3.241	2.592	0.980	2.145	2.904	3.279	*p* < 0.01

**Table 4:** Descriptive statistics and results of Mann–Whitney U tests of the HRV features of drivers with different mental states in the male group.

Features	Alert (N = 130)					Fatigued (N = 308)					Alert vs. Fatigued
	Mean	SD	Q1	Med	Q3	Mean	SD	Q1	Med	Q3	Mann–Whitney U Test
$M_{RR}$	815.9	149.1	694.8	836.5	955.5	907.0	141.8	826.1	917.8	983.3	*p* < 0.01
$D_{RR}$	49.26	26.11	30.39	45.08	59.58	74.27	29.33	53.4	72.52	90.07	*p* < 0.01
$M_{HR}$	76.0	13.77	62.8	71.73	86.35	67.86	11.12	61.02	65.37	72.63	*p* < 0.01
$D_{RMS}$	35.78	24.37	17.75	34.93	44.04	46.39	26.46	34.41	41.44	49.05	*p* < 0.01
$N_{NN15}$	20.88	22.82	1.0	15.0	34.0	28.8	20.58	15.0	27.0	35.5	*p* < 0.01
$p_{NN15}$	16.73	19.57	0.565	12.25	25.0	23.93	19.19	12.2	21.37	27.88	*p* < 0.01
$P_{LF(abs)}$	950.4	951.5	260.1	638.6	1289.0	1962.0	1634.0	722.4	1610.0	2691.0	*p* < 0.01
$P_{HF(abs)}$	600.8	903.0	104.3	262.3	666.0	850.9	1165.0	255.6	468.2	769.0	*p* < 0.01
$P_{LF(nu)}$	67.27	16.31	54.25	68.14	80.34	73.14	15.59	64.39	77.89	84.83	*p* < 0.01
$P_{HF(nu)}$	32.57	16.24	19.62	31.76	45.72	26.7	15.44	15.07	22.0	35.57	*p* < 0.01
$r_{LF/HF}$	3.225	2.831	1.187	2.146	4.096	4.226	3.304	1.81	3.54	5.631	*p* < 0.01
$P_{tot(abs)}$	1660.0	1764.0	405.6	1108.0	2026.0	3110.0	2641.0	1288.0	2474.0	4068.0	*p* < 0.01
$L_{SD1}$	25.4	17.32	12.59	24.81	31.27	32.94	18.81	24.43	29.43	34.82	*p* < 0.01
$L_{SD2}$	64.39	33.45	39.92	58.09	76.95	99.09	38.45	70.1	94.48	122.1	*p* < 0.01
$r_{SD2/SD1}$	2.986	1.13	2.286	2.716	3.359	3.37	0.979	2.673	3.357	4.08	*p* < 0.01
$E_{AP}$	0.8184	0.106	0.7485	0.8289	0.8977	0.7797	0.07939	0.7267	0.7819	0.8324	*p* < 0.01
$E_{Samp}$	1.535	0.2619	1.405	1.546	1.699	1.437	0.3083	1.205	1.432	1.624	*p* < 0.01
$\alpha_{1}$	1.234	0.2594	1.053	1.235	1.422	1.337	0.2365	1.198	1.378	1.52	*p* < 0.01
$\alpha_{2}$	0.8676	0.2441	0.6827	0.8467	1.039	0.9009	0.2553	0.7314	0.8795	1.061	*p* = 0.1647
$D_{2}$	1.935	1.357	0.6058	1.686	3.183	2.358	1.097	1.709	2.785	3.184	*p* = 0.0368

**Table 5:** Descriptive statistics and results of Mann–Whitney U tests of the HRV features of drivers with different mental states in the female group.

Features	Alert (N = 142)					Fatigued (N = 330)					Alert vs. Fatigued
	Mean	SD	Q1	Med	Q3	Mean	SD	Q1	Med	Q3	Mann–Whitney U Test
$M_{RR}$	788.2	119.2	686.2	798.2	858.8	835.3	127.7	750.1	819.1	864.7	*p* < 0.01
$D_{RR}$	41.6	10.66	33.39	40.17	48.31	54.49	14.27	44.66	52.02	62.73	*p* < 0.01
$M_{HR}$	77.8	11.32	69.86	75.17	87.44	73.4	10.52	69.39	73.25	79.99	*p* < 0.01
$D_{RMS}$	32.89	16.41	23.18	27.45	38.09	40.41	20.44	25.53	36.56	43.17	*p* < 0.01
$N_{NN15}$	18.3	20.16	5.0	9.0	29.0	27.39	22.19	7.0	24.5	37.0	*p* < 0.01
$p_{NN15}$	13.51	17.43	2.841	5.671	19.86	21.25	20.48	4.79	16.61	25.9	*p* < 0.01
$P_{LF(abs)}$	526.5	377.1	236.3	440.5	691.1	897.3	672.8	451.9	722.2	1050.0	*p* < 0.01
$P_{HF(abs)}$	442.7	419.3	189.0	305.1	509.9	655.0	581.0	280.7	461.8	744.8	*p* < 0.01
$P_{LF(nu)}$	53.95	21.32	35.86	52.15	69.41	58.93	19.41	46.4	61.37	74.55	*p* < 0.01
$P_{HF(nu)}$	45.96	21.31	30.53	47.83	64.05	41.0	19.4	25.42	38.59	53.38	*p* < 0.01
$r_{LF/HF}$	2.416	3.535	0.5599	1.09	2.274	2.174	1.839	0.8724	1.59	2.932	*p* < 0.01
$P_{tot(abs)}$	1031.0	609.2	573.4	891.2	1243.0	1682.0	1012.0	939.2	1421.0	2182.0	*p* < 0.01
$L_{SD1}$	23.34	11.66	16.45	19.48	27.02	28.69	14.53	18.12	25.94	30.65	*p* < 0.01
$L_{SD2}$	53.18	13.24	45.03	51.91	60.92	70.36	18.84	57.31	67.09	79.56	*p* < 0.01
$r_{SD2/SD1}$	2.584	0.9251	1.959	2.4	3.176	2.859	1.076	2.163	2.789	3.625	*p* < 0.01
$E_{AP}$	0.817	0.09752	0.7616	0.8178	0.8849	0.798	0.09007	0.7577	0.8083	0.8619	*p* = 0.047
$E_{Samp}$	1.611	0.2827	1.4	1.61	1.789	1.553	0.3246	1.342	1.546	1.743	*p* = 0.0661
$\alpha_{1}$	1.063	0.3163	0.8054	1.015	1.272	1.145	0.2825	0.9858	1.183	1.338	*p* < 0.01
$\alpha_{2}$	0.8886	0.2662	0.7071	0.8863	1.059	0.8854	0.2385	0.7325	0.891	1.03	*p* = 0.8787
$D_{2}$	2.264	1.103	1.211	2.485	3.252	2.809	0.7983	2.426	3.003	3.354	*p* < 0.01

**Table 6:** Comparison between our previous studies and this study considering significant differences in HRV features between the two mental states and the change tendency of HRV features between the alert and fatigued states.

Features	Sex-Independent Group			Male Group			Female Group		
	Previous Study	This Study	Accordance	Previous Study	This Study	Accordance	Previous Study	This Study	Accordance
$M_{RR}$	* ↑	* ↑	Y	* ↑	* ↑	Y	NS	* ↑	-
$D_{RR}$	* ↑	* ↑	Y	* ↑	* ↑	Y	* ↑	* ↑	Y
$M_{HR}$	* ↓	* ↓	Y	* ↓	* ↓	Y	NS	* ↓	-
$D_{RMS}$	* ↑	* ↑	Y	* ↑	* ↑	Y	NS	* ↑	-
$P_{LF(abs)}$	* ↑	* ↑	Y	* ↑	* ↑	Y	* ↑	* ↑	Y
$P_{HF(abs)}$	* ↑	* ↑	Y	NS	* ↑	-	NS	* ↑	-
$P_{LF(nu)}$	NS	* ↓	-	NS	* ↓	-	NS	* ↓	-
$P_{HF(nu)}$	NS	* ↑	-	NS	* ↑	-	NS	* ↑	-
$r_{LF/HF}$	NS	* ↑	-	NS	* ↑	-	NS	* ↑	-
$P_{tot(abs)}$	* ↑	* ↑	Y	* ↑	* ↑	Y	* ↑	* ↑	Y
$L_{SD1}$	* ↑	* ↑	Y	* ↑	* ↑	Y	NS	* ↑	-
$L_{SD2}$	* ↑	* ↑	Y	* ↑	* ↑	Y	* ↑	* ↑	Y
$r_{SD2/SD1}$	* ↑	* ↑	Y	NS	* ↑	-	NS	* ↑	-
$E_{AP}$	NS	* ↓	-	NS	* ↓	-	NS	NS	-
$E_{Samp}$	NS	* ↓	-	NS	* ↓	-	NS	NS	-
$\alpha_{1}$	* ↑	* ↑	Y	NS	* ↑	-	* ↑	* ↑	Y
$\alpha_{2}$	NS	NS	-	NS	NS	-	NS	NS	-
$D_{2}$	* ↑	* ↑	Y	NS	NS	-	* ↑	* ↑	Y

Abbreviations: * denotes *p* < 0.01; NS denotes not significant; ↑ denotes a significant increase from the alert state to the fatigued state; ↓ denotes a significant decrease from the alert state to the fatigued state; Y indicates whether the significance of the difference in HRV features between the two mental states in our previous study and this study are/are not identical with/without the same change tendency if *p* < 0.01.

**Table 7:** Performance of fatigue detection classifiers for the three driver groups based on the best subsets of 2, 3, and 4 features.

Number of Features Used	Group	Subset of Features	SEN	SPE	PPV	ACC
2	Sex-independent group	$p_{NN15}$ $,\;r_{SD2/SD1}$	93.1%	56.5%	85.8%	83.5%
	Male group	$M_{RR}$ $,\;r_{SD2/SD1}$	96.5%	83.3%	91.7%	92.0%
	Female group	$D_{RR}$ $,\;P_{LF(abs)}$	98.1%	66.7%	86.9%	88.5%
3	Sex-independent group	$M_{RR}$ $,\;N_{NN15}$ $,\;r_{SD2/SD1}$	89.0%	79.6%	91.7%	86.3%
	Male	$P_{HF(nu)}$ $,\;\alpha_{2}$ $,\;D_{2}$	100.0%	73.7%	92.1%	93.5%
	Female	$M_{RR}$ $,\;p_{NN15}$ $,\;\alpha_{1}$	93.9%	84.6%	92.0%	90.7%
4	Sex-independent group	$M_{RR}$ $,\;M_{HR}$ $,\;N_{NN15}$ $,\;r_{SD2/SD1}$	89.0%	79.6%	91.7%	86.3%
	Male group	$P_{HF(abs)}$ $,\;L_{SD2}$ $,\;E_{Samp}$ $,\alpha_{1}$	98.2%	85.7%	94.8%	94.8%
	Female group	$M_{RR}$ $,\;p_{NN15}$ $,\;r_{SD2/SD1}$ $,\alpha_{1}$	98.0%	80.8%	90.6%	92.0%

## Data Availability

The data presented in this study are available from the corresponding author on request.
